# Can Moderate Levels of Organic Selenium in Dairy Cow Feed Naturally Enrich Dairy Products?

**DOI:** 10.3390/ani10122269

**Published:** 2020-12-01

**Authors:** Irene Azorín, Josefa Madrid, Silvia Martínez, Marina López, María Belén López, Miguel José López, Fuensanta Hernández

**Affiliations:** 1 Department of Animal Production, Faculty of Veterinary, Regional Campus of International Excellence (CEIR) Campus Mare Nostrum, University of Murcia, 30100 Murcia, Spain; irene.azorin1@um.es (I.A.); silviamm@um.es (S.M.); marina.lopez9@um.es (M.L.); mjlopeza@um.es (M.J.L.); nutri@um.es (F.H.); 2 Department of Food Technology, Nutrition and Bromatology, Faculty of Veterinary, Regional Campus of International Excellence (CEIR) Campus Mare Nostrum, University of Murcia, 30100 Murcia, Spain; mbelen@um.es

**Keywords:** organic selenium, feed additive, milk, dairy

## Abstract

**Simple Summary:**

Milk and dairy products, such as cheese and yogurt, can be useful sources of selenium-enriched products to increase human Se intake. We studied the effect of dairy cow feed supplementation with inorganic plus organic Se on milk yield, and on the Se enrichment of milk and dairy products to obtain naturally enriched products. Two groups of lactating cows were assigned to two feeding treatments, both with 0.240 mg Se/kg of ration dry matter: One was supplemented with inorganic Se, the other with a 60/40 ratio of inorganic Se/organic Se. The results showed that the inclusion of inorganic plus organic Se did not affect the yield or basic chemical composition of milk; however, the Se content of milk was higher with inorganic plus organic Se supplementation. Cheese from cows fed inorganic plus organic Se had a higher Se content, although this effect was not observed for yogurt. At a moderate level in the diet, sodium selenite plus Se yeast may be more effective than only inorganic Se, increasing the Se concentration in milk and cheese.

**Abstract:**

This work studied the effect of dairy cow ration supplementation with inorganic plus organic Se on metabolic status, milk yield, and the quality of milk and dairy products, especially its Se content. Twenty multiparous Holstein Friesian lactating cows were assigned to two feeding treatments. The cows were fed with 22.5 kg dry matter (DM) of total mixed ration (11.75 kg DM of forage plus 10.75 kg DM of concentrate) by head. There were two different concentrates with the same Se content (0.240 mg/kg of ration DM) but with different Se sources: The control (CON) was supplemented with inorganic Se (sodium selenite); and the other (IOSe) was supplemented with sodium selenite plus organic Se (Sel-Plex^®^), at 0.144 and 0.096 mg Se/kg of ration DM, respectively. The results indicated that, in general, the IOSe treatment did not modify the metabolic profile, and even decreased the total oxidant status (*p* < 0.05) and did not lead to a deterioration of quality and yield of milk. However, milk and cheese from IOSe had higher Se content (an increase of 29.7% and 38.2%, respectively) than CON (*p* < 0.01), but this effect was not observed in yogurt. In general, physical or sensorial parameters of cheeses did not show differences between treatments. Moderate inorganic plus organic Se supplementation may be more effective than inorganic Se, increasing the Se content in milk and cheese, without causing a deterioration in quality or productive parameters.

## 1. Introduction

Selenium is an essential micronutrient required in trace quantities for normal growth and development of animals, which plays an important role in many metabolic functions related to antioxidant activity and the prevention of degenerative processes [1]. This element is essential to the functionality of many selenium dependent enzymes such as glutathione peroxidase (GSH-Px), thioredoxin reductase, or iodothyronine deiodinase [1,2]. Se deficiency in humans can lead to various diseases including cardiovascular disease, cancer, and immune system dysfunction [3]. Chronic selenium deficiency can lead to Keshan disease (a cardiomyopathy) and Kashin-Beck disease (an osteo-arthropathy); both disorders have been described in areas of China with a low Se content in the soil [4]. The highest levels of Se intake have been recorded in seleniferous regions of China and Venezuela, which may pose a risk of Se toxicity [5]. Indeed, the entrance of selenium to the human organism mainly occurs through the intake of water and food [1], which is linked to the concentration of Se in the soil. The adult human recommended dietary allowance of Se is 55 μg/day [6], and the highest tolerable intake level has been set at 400 μg/day [7]. Se deficiencies are widespread among people all over the world, especially in areas with poor selenium soils. Fairweather-Tait et al. [5] collected data regarding Se intake from some several geographic areas and found high global variability, as well as large geographic areas lacking data. For some European countries, these authors indicated Se mean intakes <50 μg/day. In addition, Se status in animals and humans is usually assessed by the determination of the total selenium in blood and by determination of the GSH-Px [8], and the World Health Organization (WHO) [7] recommends a minimum intake to achieve two-thirds of the optimal activity of GSH-Px. Stoffaneller and Moser [9] reported that suboptimal Se status is widespread throughout Europe and the Middle East.

In situations of Se deficiency, one option would be to improve the selenium content of foods. Particularly, an opportunity in the livestock sector may be to increase Se levels in animal origin foods, without resorting to extra manipulations after its production. It is known that Se is one of the minerals with the best responsiveness of milk of dairy cows to dietary supplementation [10]. Moreover, milk and dairy products, such as cheese and yogurt, are usually present in the human diet and are regularly consumed [11]. The use and optimization of both strategies could generate animal products naturally enriched in Se easily accepted by the population.

The bioavailability of Se strongly depends on the chemical form found in the diet [12]. Selenium supplements exist in two main forms: Inorganic mineral salts such as sodium selenate and sodium selenite; and organic forms such as selenomethionine (SeMet, predominant form in Se enriched yeast). Mehdi et al. [13] found that SeMet can be accumulated in the body as methionine amino acid of proteins, and serves as a reserve of Se, which ensures good Se status. However, the common form used in dairy cow supplement is sodium selenite because it is less expensive than organic selenium. However, organic selenium forms are more bioavailable and safer than inorganic forms when improving the selenium status of ruminants, and might be transferred more readily to milk [14,15,16,17].

Accordingly, there is considerable interest in supplementing the diets of lactating animals with organic Se, and there are many studies that show positive results with increased levels of Se in milk [18,19,20,21] and dairy products [22,23,24]. However, most studies used organic Se levels higher than those authorized in the European Union (maximum of 0.2 mg of organic Se/kg of total ration, and total Se (organic + inorganic) cannot exceed of 0.5 mg/kg of total ration) [25]; only Ling et al. [24] studied an Se supplementation within the limits of this legislation, but they used levels close to the maximum.

Recent studies indicate that the supplementation of the diets of dairy cattle with organic Se could affect the aromatic profile of milk and derived dairy products such as cheese [26,27]. In addition, the nutritional composition and organoleptic characteristics of Se enriched dairy products could be modified by technological processing [28,29].

As a consequence, the hypothesis of this work was that the supplementation of lactating cow feed with inorganic plus organic Se at moderate levels would improve the Se status of the animal—the concentration of Se in milk and dairy products—without negatively affecting their nutritional or organoleptic properties. The aim of this work was to study the effect of sodium selenite plus Se yeast supplementation on feeding dairy cows’ (within the levels of European Union legislation and with a wide safety margin) health status, yield, milk composition, and the chemical and technological quality of dairy products, in order to obtain enriched products for the market.

## 2. Materials and Methods

The experimental procedure was performed according to the protocol approved (A13170805) by the Ethics Committee of the University of Murcia and the Authorities of the Region (Murcia, Spain), according to the European Union regulation (2010/63/EU Directive) related to the protection of animals used for scientific purposes [30].

### 2.1. Animals and Experimental Design

The trial was conducted over 2 months at the Dairy Cattle Unit of Veterinary Teaching Farm (University of Murcia). Twenty multiparous Holstein Friesian lactating cows were used in the experiment. Animals had an average weight of 649.7 ± 48.55 kg LW, 170.2 ± 33.15 days in milk, and a 30.0 ± 4.85 kg/day of milk yield. They were randomly distributed into two groups of 10 cows in each. Thus, the cows were housed in two pens separated by an electric fence, with a straw-covered floor, provided with feeders for total mixed rations (TMR), and with free access to water.

For the experiment, each group of cows was assigned to a feeding treatment: One was a control, supplemented with inorganic Se (CON); the other was supplemented with inorganic and organic Se (IOSe). Both TMR diets had the same base diet (Table 1), with a forage-to-concentrate ratio of 52.25:47.75. In the CON treatment, a source of inorganic Se (as sodium selenite) (0.240 mg/kg dry matter (DM) of TMR) was used; and in the IOSe treatment, 60% of Se (0.144 mg/kg DM of TMR) was incorporated as inorganic Se (sodium selenite), and 40% (0.096 mg/kg DM of TMR) as organic Se using Sel-Plex^®^ (Alltech, Nicholasville, KY, USA). This additive is rich in organic Se derived from a specific strain of *Saccharomyces cerevisiae,* containing >63% selenomethionine (SeMet) (CNCM I-3060).

The estimated Se levels were similar in both diets, and <55% of the maximum level of Se in the ration according to the legislation of the European Union. Regulation 2019/804 for CNCM I-3060 [25] admits a maximum level of organic Se of 0.2 mg Se/kg diet, and a total Se of 0.5 mg/kg diet in a complete feed with a moisture content of 12%. Both diets were formulated to be iso-energetic and iso-nitrogenous, according to nutritional requirements for mid-lactation cows of the Fundación Española para el Desarrollo de la Nutrición Animal (FEDNA) [31] The chemical composition of ingredients and experimental rations are presented in Table 2. The TMR was offered twice daily after each milking, distributing 22.5 kg DM of TMR per head daily.

Cows were milked twice a day at 7:00 h and 19:00 h; and on days 0, 21, and 49, the individual milk yield was recorded (T_0_, T_21_ and T_49_, respectively), and individual milk samples (representatives of the morning and afternoon milking) were also taken. Samples were divided into two subsamples: One (approximately 150 mL) for pH determination, and fat, protein, and lactose analysis; the other (approximately 100 mL), which was stored at −20 °C, was analyzed for mineral content (Ca, P, Zn, Cu, and Se) of the milk.

Blood samples of all cows were collected via coccygeal venipuncture before morning feeding on days 0, 21, and 49 of the experiment. Two samples per animal of whole blood were taken using Lithium-heparin tubes (VACUETTE^®^, Greiner Bio-One International GmbH, Kremsmünster, Austria): One sample was used to analyze the mineral content (Ca, P, Zn, Cu, and Se) and the GSH-Px activity of the whole blood; and the other sample was centrifuged (3000× *g* for 15 min) to obtain plasma and stored at −80 °C until metabolic profile determination was carried out. A sample of whole blood per animal was sampled with K_3_EDTA tubes (VACUETTE^®^, Greiner Bio-One International GmbH, Kremsmünster, Austria) to analyze hemoglobin (Hb).

### 2.2. Yogurt Elaboration

Yogurts were prepared on three different days, after 49 day of the experiment, (49, 52, and 55 day) at the Food Technology Pilot Plant of the University of Murcia. Each day, six yogurts were prepared per treatment, 3 were used for physicochemical analysis, and 3 for sensorial study. One liter of pasteurized milk (95 °C during 240 s) was allocated in a stainless-steel vat with 100 g of milk powder and 20 g of sugar, mixing and heating until 95 °C. Then, it was left to temper at 45 °C, and *Streptococcus thermophilus* and *Lactobacillus bulgaricus* ferment was added (YOMIX-300, Chr-Hansen, Madrid, Spain) in a proportion of 1 g/100 L. Finally, yogurts were packaged and incubated at 45 °C during 4 h and kept at 4 °C after incubation process.

### 2.3. Cheese Elaboration

Also, cheeses were prepared on three different days, after 49 d of the experiment, (50, 53, and 56 d) at the Food Technology Pilot Plant of the University of Murcia. Each day three cheeses were prepared per treatment. Ten liters of pasteurized milk (75 °C during 20 s) were allocated in a 12 L double 0 stainless-steel vat (Pierre Guerin Technologies, Mauzé, France), and tempered for 10 min until a constant temperature of 33–34 °C. In stirring, 0.3 mL/kg of anhydrous CaCl_2_ (Chr. Hansen, France) and 0.3 mL/kg of calf rennet (80% chymosin; 145 IMCU/mL) supplied by Caglio Star España, S.A. (Murcia, Spain) were added. It was left to rest for 40 min. After that time, it was cut for 3 min with a speed of 25%, leaving to stand for 5 min. Then, 80 g of salt was added and stirred again for 1 min. Following stirring, it was left to rest for a further 10 min and stirred again for 1 min. Finally, the whey was drained, and the curd was filled into molds without pressing. The cheese was left draining in refrigeration at 4 °C during 24 h. Each cheese was split into two halves, one for physicochemical analysis and the other for sensorial study.

### 2.4. Laboratory Procedures

#### 2.4.1. Feed Analysis

Forages (alfalfa hay and barley straw), concentrates, and TMR (CON and IOSe) were weekly sampled. TMR samples were pre-dried in a convection oven at 60 °C for 48 h. All samples were ground to pass a 1 mm screen (Retsch ZM 200 Ultra Centrifugal Mill; Retsch, Hann, Germany). Feed samples were analyzed by the Association of Official Analytical Chemists (AOAC) procedures [33]: dry matter by AOAC 934.01 method, ash content by AOAC 942.05 method, ether extract (EE) by AOAC 920.39 method, and crude protein (CP) by AOAC 2001.11 method. Neutral detergent fiber (NDF) and acid detergent fiber (ADF) were analyzed by the Van Soest et al. [34] methods, and acid detergent lignin (ADL) was determined through the solubilization of cellulose with 72% sulphuric acid.

#### 2.4.2. Mineral Analysis (Ca, P, Zn, Cu, and Se)

Samples of feeds, whole blood, milk, and dairy products were digested in a microwave digestion system (Milestone Ethos X Microwave, Sorisole, Italy) in the presence of HNO_3_, and Se and other minerals (Ca, P, Zn, and Cu) were determined by inductively coupled plasma-mass spectrometry (Agilent 7900 ICP-MS, Santa Clara, CA, USA) using the method of standard addition.

#### 2.4.3. GSH-Px, Hb and Metabolic Profile

GSH-Px activity and Hb were analyzed in whole blood samples. GSH-Px activity was determined with a commercial kit (Ransel test kit, Randox Laboratories Ltd., Crumlin, UK) adapted from the method of Paglia and Valentine [35]. This technique is based on the reaction of oxidation of glutathione (GSH) by cumene hydroperoxide catalyzed by GSH-Px, and the transformation from oxidized GSH form to reduced form with an associated oxidation reaction of NADPH to NADP^+^, in occurrence with glutathione reductase and NADPH. The rate of reduced GSH formation is monitored by the decreasing of absorbance produced by the consumption of NADPH. Hb was analyzed by an automated hematological analyzer (Advia 120, Siemens Healthcare Diagnostics SL, Barcelona, Spain).

The general metabolic profile evaluation in plasma samples (glucose, total cholesterol, total proteins, triglycerides (TG) and urea) was analyzed. These assays were carried out on an automated chemistry analyzer (Olympus AU600, Olympus Diagnostica Europe GmbH, Ennis, Ireland). For these assays commercial Beckman kits (Beckman Coulter Inc., Fullerton, CA, USA) were used. Thus, glucose was measured by the hexokinase G-6-PDH method; total cholesterol was determined by the cholesterol dehydrogenase method; total proteins were measured by the adapted Weichselbaum method [36]; total triglycerides were hydrolyzed by a combination of microbial lipases to give glycerol and fatty acids, to be determined; and urea was measured by the enzymatic adapted Talke and Schubert method [37].

The β-hydroxybutyrate (β-BHA) was determined by commercial Randox kit (Ranbut assay, Randox laboratories Ltd., Crumlin, UK). This method is based on the oxidation of β-BHA to acetoacetate by the enzyme β-BHA dehydrogenase, where concomitantly the NAD^+^ is reduced to NADH, of which absorbance changes are directly related with the β-BHB concentration Plasma non-esterified fatty acids (NEFAS) was quantified by commercial Randox kit (Randox laboratories Ltd., Crumlin, UK). This NEFAS assay protocol is based on a colorimetric method associated to enzymatic incubation with acyl-CoA-synthetase, acyl-CoA-oxidase, and peroxidase.

In addition, TAC (antioxidant capacity) and TOS (oxidative status) were analyzed in plasma by the methods described by Erel [38,39]. TAC protocol is based on decolorization of 2,2’-azinobis-(3-ethylbenzothiazoline-6-sulfonic acid) radical cation (ABTS^•+^) performed by antioxidants present in the sample. TOS procedure is based on the oxidation of ferrous ion to ferric ion in the presence of oxidant species of samples, thus ferric ion making a colored complex with xylenol orange in acidic medium, that can be measured spectrophotometrically.

#### 2.4.4. Proximate Composition and pH of Milk and Dairy Products

The proximate composition of milk (fat, protein and lactose) and yogurt (fat and protein) were analyzed by infrared spectroscopy (MilkoScan FT6000, Foss Electric, Hillerod, Denmark) according to IDF standard 141B:1996 [40]. In cheeses, chemical composition (dry extract, fat, and protein) was determined as following: Dry matter was measured in grated cheese samples (3 g ± 10 mg), which were dried to constant weight according to IDF Standard 4:2004 [41]; fat content of cheeses was determined by the IDF Standard 5:2004 procedure [42], and protein content was determined by Kjeldahl method (IDF Standard 25:2008) [43].

pH was directly determined in milk and yogurt. On cheese samples, pH measurements were made in grated cheese (5 g ± 0.1 mg) suspended in 30 mL of distilled water and stirred for 10 min. The measurements were carried out using a Crison^®^ pH meter (micro pH 2001, Barcelona, Spain) connected to a Crison^®^ glass combined electrode (1952–2002) previously calibrated at room temperature.

#### 2.4.5. Physical Parameters of Dairy Products

Yogurt syneresis (released whey) was measured after weighing 50 g of yogurt sample and centrifuging it at 3000 rpm for 20 min in order to subsequently measure the amount of whey drained, used as an index of syneresis. Cheese yield was calculated as the amount of cheese expressed in kilograms obtained from 100 kg milk. The color/lightness attributes were determined in dairy products with a CR-400 MINOLTA colorimeter (Konica Minolta Sensing, Ramsey, NJ, USA). An average of three L* (lightness), a* (redness), and b* (yellowness) readings was taken per replication.

Texture profile analysis (TPA) was performed on cheeses using a texture analyzer (TA-XT Plus, Stable Micro Systems Ltd., Godalming, UK) equipped with a load cell of 500 N. The analyses were conducted in triplicate on cheese cube shaped samples (3 cm^3^) rindless, and at room temperature (20 °C). For TPA tests, each sample was compressed twice to a distance of 15 mm using a P/100 probe (compression platen of 100 mm of diameter) moving at a speed of 1 mm/s. The software Exponent Lite (version 5.1.1.0, Stable Micro Systems Ltd., Godalming, UK) was used and the texture parameters determined were hardness (expressed as N), cohesiveness (dimensionless), gumminess (product of hardness and cohesiveness, expressed as N), elasticity (expressed as mm), chewiness (product of gumminess × elasticity, expressed as N mm), and adhesiveness (expressed as N s), calculated as described by Bourne [44].

#### 2.4.6. Sensorial Parameters of Dairy Products

A sensory analysis of cheese and yogurt was performed by 10 trained panelists, repeated over three independent sessions. They were specifically trained to determine the sensory attributes. For cheeses, the attributes analyzed were odor, cream odor, cow milk flavor, cream flavor, salty, firmness, granularity, juiciness, and fatty; and for yogurts, the attributes determined were odor, acid odor, cow milk odor, acid flavor, cow milk flavor, cremosity, metallic flavor, astringency, and consistency. Half of each cheese was divided into wedges of approximately 1 cm thickness, while yogurts were presented in individual plastic cups. Samples were labeled using randomly chosen digits. The descriptive sensory analyses were performed using a scoring system with a structured intensity scale, ranging between 1 and 9 for cheese, and between 0 and 10 for yogurt. Unsalted crackers and mineral water were served to remove any aftertaste between samples.

### 2.5. Statistical Analyses

Data of whole blood, plasma, and milk were analyzed using a general linear model with repeated measures in IBM SPSS Statistics software (IBM Corporation, Armonk, NY, USA), where the type of treatment (CON or IOSe) was considered as an inter-subject factor and the time of measure at different days of trial (T_0_, T_21_, and T_49_) was considered as an intra-subject factor. For cheese and yogurt, physicochemical data were analyzed using the mixed procedure of the same statistics software, considering the type of treatment as a fixed effect, and the elaboration batch as a random effect. Student’s *T* tests were used for sensorial analysis using the same software. Results were considered statistically significant at at *p* < 0.05.

## 3. Results

### 3.1. Whole Blood and Plasma Analyses

The effect of feeding inorganic plus organic selenium supplement on the mineral (Se, P, Ca, Cu, and Zn) content of the whole blood of dairy cows at 0, 21, and 49 days of experiment are presented in Table 3. Mineral content (including selenium) in whole blood was not affected (*p* > 0.05) by the treatment, except for zinc levels, which were lower in the blood of CON cows. In addition, hemoglobin and GSH-Px activity were unaffected by treatment (*p* > 0.05). However, GSH-Px activity was increased (*p* < 0.001) throughout the trial (21,274, 24,316, and 26,631 U/L; 196.2, 228.7, and 256.0 U/g Hb, for T_0_, T_21_, and T_49_, respectively). No interactions between treatment and time were found (*p* > 0.05) on these parameters.

Table 4 presents the effect of feeding inorganic plus organic selenium supplement on the biochemical profile and oxidative markers in the plasma of dairy cows during the experiment until day 49. A significant increase (*p* < 0.001) of the cholesterol concentration (226.9, 262.1, and 274.1 mg/dL, for T_0_, T_21_, and T_49_, respectively) and significant decrease (*p* < 0.01) of the β-hydroxybutyrate (0.392, 0.389 and 0.318 mmol/L, for T_0_, T_21_ and T_49_, respectively) were observed throughout the experiment. An effect of the type of ration (*p* < 0.05) and an interaction between the time and ration (*p* < 0.01) were found for β-hydroxybutyrate, with lower values in the CON group. In addition, the TAC was not modified by the treatment, while TOS was lower (*p* < 0.05) with IOSe diet. Other effects were not found for the parameters analyzed in the plasma.

### 3.2. Yield, Composition and pH of Milk

The effect of feeding with an inorganic plus organic selenium supplement on milk yield, composition, and pH of milk of dairy cows at 0, 21, and 49 days of experiment are presented in Table 5. No differences were found (*p* > 0.05) in milk yield between treatments. In addition, the fat, protein, and lactose content, and pH of milk were unaffected (*p* > 0.05) by inorganic plus organic selenium supplementation. Regarding the composition of the mineral fraction of milk, no treatment effects were found (*p* > 0.05), except for the concentration of selenium in milk, which was higher (*p* < 0.01) in the IOSe group compared to the CON group (with overall mean values of 1.79 versus 1.38 µg/100 g, respectively). However, a day effect (*p* < 0.05) was observed for fat, protein, lactose, Zn, and Cu content, showing fluctuations throughout the trial in both treatments. In addition, no interactions between treatment and time were found (*p* > 0.05) on the yield, composition, or pH of milk.

### 3.3. Physicochemical Composition and Sensory Parameters of Yogurt

Table 6 presents the effect of feeding an inorganic plus organic selenium supplement on the physicochemical composition of yogurt. No differences were found (*p* > 0.05) between treatments on proximate composition, pH, mineral content (including selenium), syneresis, or colorimetric parameters of the yogurt. Only the Ca content was slightly higher (*p* < 0.05) in the CON treatment.

**Figure 1:**
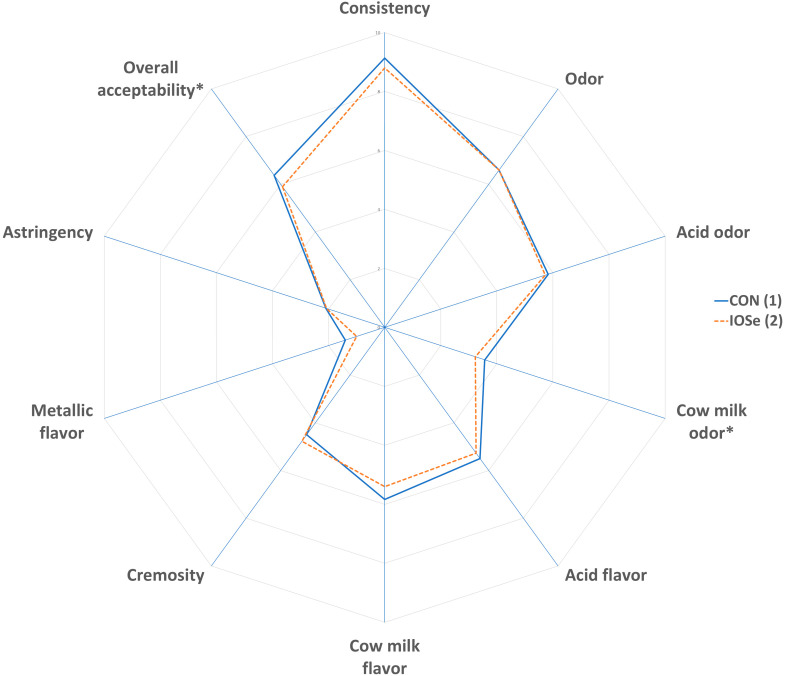
Effect of feeding cows with different of sources of selenium on sensory parameters of yogurts. Spider chart: (1) Treatment: total mixed ration with inorganic Se. (2) Treatment: Total mixed ration with inorganic plus organic Se. The asterisk (*) indicates that there are differences (*p* < 0.05) between treatments.

The results of the panelists’ evaluation of yogurt are shown in Figure 1. Significant differences (*p* < 0.05) were found in the odor of cow’s milk, which was slightly lower in IOSe yogurt. In addition, the overall acceptability showed differences (*p* < 0.05) between treatments. Thus, the CON group yogurt obtained higher evaluation results than the IOSe group yogurt. The other parameters evaluated were not affected (*p* > 0.05) by inorganic plus organic selenium supplementation.

### 3.4. Physicochemical Composition and Sensory Parameters of Fresh Cheese

Table 7 presents the effect of feeding inorganic plus organic selenium supplement on the physicochemical composition of cheeses. No differences were found between treatments (*p* > 0.05) on proximate composition, except for the dry extract (*p* < 0.05) that was higher in the CON group. In terms of mineral composition of cheese, Ca, P, and Zn content was not modified by treatments (*p* > 0.05); but level of Cu showed greater level (*p* < 0.001) in CON cheese, and Se content was greater (*p* < 0.01) in IOSe than CON cheese (8.29 versus 6.00 μg/100 g, respectively). In addition, no effects of Se source were observed in cheese yield, colorimetric parameters, and texture profile (*p* > 0.05), except for elasticity (*p* < 0.01), which was slightly lower in CON cheese.

**Figure 2:**
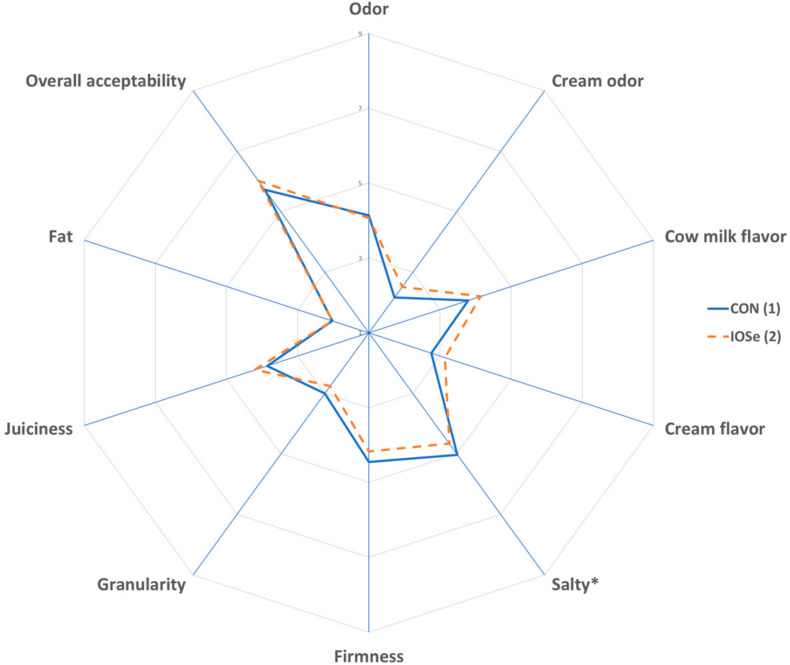
Effect of feeding of cows with different of sources of selenium on sensory parameters of cheeses. Spider chart: (1) Treatment: Total mixed ration with inorganic Se. (2) Treatment: Total mixed ration with inorganic plus organic Se. The asterisk (*) indicates that there are significant differences (*p* < 0.05) between treatments.

The results of the sensory profile of cheeses are shown in Figure 2. A significant difference was found (*p* < 0.05) in the salty taste of cheese, it being greater in CON cheese; the other sensory parameters of cheese were unaffected by treatments (*p* > 0.05). In addition, there were no differences (*p* > 0.05) in the overall acceptability of CON and IOSe cheese.

## 4. Discussion

The Se composition of the rations, both in the CON and IOSe groups, were close to estimated values (0.240 mg Se/kg DM), with deviations of ≤15%.

Except for the level of Zn, the mineral content of whole blood was not affected by dietary treatments, and the Zn levels were within the physiological range for cattle [45]. All animals had a mean concentration of Se in blood higher than 120 µg Se/L, indicating an adequate selenium status. Stowe and Herdt [46] considered that at least 100 µg Se/L in whole blood is required to achieve an optimal immune capacity and fertility, and Smith et al. [47] even recommended 200 µg Se/L to achieve optimal resistance against infectious mastitis.

Similar to our study, Juniper et al. [48] observed that Se sources in the diet did not significantly affect Se concentrations in the whole blood of cows. Different results were obtained by Calamari et al. [20], who found a Se source effect with greater values of total Se in blood with an organic Se dietary treatment comparing with selenite dietary treatment; in addition, Weiss [49] reported an increase of 18% in the whole blood Se concentration in animals fed a diet including organic Se. This pattern was reported in studies in which the total dietary selenium intake in the control group was relatively low. However, the lack of response in the current trial may be associated with the fact that the total dietary selenium intakes of 5.4 mg/day per animal in the control and experimental treatment were above the threshold at which a response would be recorded. Knowles et al. [50] indicated that the level of Se in blood per milligram of Se administered could depend on Se intake, at which the mechanisms of absorption and distribution of dietary Se into blood are saturated; they found that a total dietary intake of 2 mg Se/day with selenized yeast was more efficient than with 4 mg/day.

In addition, Se can only be incorporated into red blood cells during erythropoiesis [51], and because this process occurs slowly, there will be a lag in blood Se response following changes in Se supplementation. Moreover, Gong et al. [21] indicated that SeMet is deposited in erythrocytes more easily than selenite, which may explain the difference in the whole blood Se concentration between organic selenium and selenite supplementation. We would probably have found a greater response with more days of testing, since successful results in other studies were obtained with longer blood sampling periods [20,21]. However, quantitatively, the highest level of Se in blood was found on day 49 with IOSe treatment (158.6 µg/L) although statistical differences were not found.

In general, the biochemical plasma profile did not change throughout lactation, with the exception of cholesterol and β-hydroxybutyrate. These metabolic indicators are related to lipid metabolism, and it has been shown that they can vary according to the physiological state of the cow [52]. As lactation advanced cholesterol increased and β-hydroxybutyrate decreased, which may indicate an improvement in metabolic status. When there are problems in lipoprotein synthesis, triglycerides accumulate in the liver, with the consequent risk of fatty liver [53]. Cholesterol is one of the components of lipoproteins, and Rayssiguer et al. [54] indicated that a lower cholesterol concentration increases the risk of this metabolic disorder. In addition, the level of β-hydroxybutyrate has been used as a ketonemic indicator; thus, a high concentration of this ketone body is related to ketosis. McArt et al. [55] defined subclinical or clinical ketosis from 1.2 to 2.9 mmol/L, or ≥3.0 mmol/L of blood β-hydroxybutyrate, respectively. In this study, the values were under these limits, with a slightly lower mean value in the control group.

The indicators of the blood oxidative status studied showed that the whole blood GSH-Px activity, despite not being affected by treatments, increased throughout the experiment in both treatments (196.2 T_0_, 228.7 T_21_, and 256 U/g Hb at T_49_). A similar effect was reported by Gunter et al. [56], indicating a greater activity of the antioxidant enzyme GSH-Px over time of supplementation. Furthermore, the lack of effect of type of source of dietary selenium on GSH-Px activity was reported in a review by Weiss [49], in which 9 of 11 studies reported no significant difference in GSH-Px activity when comparing organic and inorganic Se supplementation. Calamari et al. [20] also reported no effect of dietary selenium source (with organic or inorganic Se supplementation), but they found differences between the supplemented and unsupplemented diets at day 42 of the experimental period. In contrast, Gong et al. [21] observed a source effect increasing GSH-Px activity in cows supplemented with organic selenium. However, the antioxidant capacity of cows (TAC) was not altered between treatments, but the oxidative status (TOS) was lower in the IOSe diet. Gong et al. [21] found that the serum malondialdehyde concentration (a marker for oxidative stress) was lower in the group supplemented with organic Se, but they also found an improvement in antioxidative parameters with organic selenium supplementation.

The absence of effects of the treatments on milk yield was in line with Juniper et al. [48] and Heard et al. [18], who observed no effect on milk yield when rations for dairy cows were supplemented with organic Se. According to Gong et al. [21] and Calamari et al. [20], the fat, protein, and lactose levels were not altered by IOSe treatment. Our results support the general view that the selenium source is unlikely to markedly affect the concentration of these components. Responses in terms of yield would be expected when Se-deficient cows are fed with a Se supplement.

In our study, the selenium milk level was higher for the IOSe ration group. This result is in accordance with the findings of Calamari et al. [20] and Stockdale and Gill [57], who found higher selenium levels in milk from cows with a diet enriched with organic selenium. Knowles et al. [50] observed the same results, with selenium concentrations in milk 1.5 to 5.5 times greater in cows supplemented with organic selenium at day 133 of the trial, and Ortman and Pehrson [58] observed an increase of selenium concentration in milk within one week of beginning organic selenium supplementation. It was suggested by Pehrson [59] that the increase of selenium concentration in milk derived from diets containing organic selenium may be due to the preferential mammary gland uptake of SeMet, which is readily incorporated into milk protein. Heard et al. [18] and other previous reports on cows [60,61] showed that selenium concentrations peaked at approximately day 7 after the introduction of Se yeast.

According to Regulation 1169/2011 (articles 32 and 33, and Annex XIII part A) of the European Union, beverages that provide 7.5% of the daily reference intake of selenium (55 µg) can be labeled as a “source of selenium” (4.13 µg/100 mL) [62]. In our experiment, the highest values were found at T_21_ and T_49_ (2.01 and 1.85 expressed as µg/100 mL, respectively) of the IOSe treatment group. Therefore, this milk could not be labeled as a source of Se, despite the fact that supplementation with inorganic plus organic selenium increased its content by 29.7%.

A day effect was observed for fat, protein, and some microelements (Zn and Cu) content in both treatment groups, with a slight decrease, although milk production was not affected. In addition, the fat/protein ratio of milk in all cases was very close to or within the ranges indicated by Čejna and Chládek [63] for states without metabolic disorders (from 1.2 to 1.4).

In general, the physicochemical composition of the yogurt was not affected by treatment; only Ca content was slightly lower in the IOSe group yogurt. On the other hand, pH values in yogurt were lower than in milk. This was an expected result, considering the lactic fermentations that occur in the preparation of yogurt. In contrast, the average selenium content of yogurt was quantitatively higher than in milk for both treatments. Csapó [64] found a slight increase of yogurt selenium content from two origins, one from dairy cattle fed on a feed supplemented with organic selenium, the other from these dairy cows before supplementation, suggesting that this effect could be explained by the water loss that occurred during the production phases, which increased the selenium content of this product. Furthermore, it should be highlighted that the rest of the minerals also had a higher concentration in yogurt.

The study of the feeding treatment effect on the sensory parameters of yogurt showed that the smell of cow milk was altered, being less strong in IOSe yogurt; this could be linked with the lower overall acceptability of IOSe enriched yogurt. Alzate et al. [29] reported that higher levels of Se in fermented milks induce a metallic odor and flavor and a pinkish color in the product, although selenium was included using sodium selenite during the factory process, and selenium levels at which this effect was observed were 50 times greater than those found in our trial. However, in our case, yogurts from both treatments attained a medium level of acceptance.

For yogurt, according to the regulation of the European Union, solid food that provide 15% of the daily reference intake of selenium (8.25 µg/100 g), could be labeled as a source of selenium. Thus, the yogurt obtained by both dietary treatments could not be labeled specifying this property.

In cheese, the general proximate composition was not affected by dietary treatments, except for the dry extract, but this did not affect the cheese yield. However, the highest levels of Se found in experimental cheese were due to the fact that Se is largely associated with the different whey and casein protein fractions of milk. Yoshida et al. [65] indicated that 40% of milk selenium is linked with the casein fraction of cow milk, while Mathias et al. [66] reported that 60% of total milk selenium was present in the milk casein fraction. Evidently, κ-casein has the highest selenium content of all the milk proteins [67]. Therefore, a great part of the selenium present in milk will be retained in the cheese curd associated with the casein micelles. Knowles et al. [50] showed that the incorporation of Se into casein derived from Se yeast was much greater than from inorganic Se, which suggests that SeMet from selenized yeast proteins can withstand rumen degradation and become available for milk protein synthesis. Thus, the average level of Se obtained in IOSe cheese was 8.29 μg Se/100 g. As indicated previously, a minimum amount of 8.25 μg Se/100 g is necessary to consider a solid food a source of Se. Therefore, cheese from the IOSe treatment group could be considered a source of this mineral. A lower Cu level was found in IOSe cheese compared with the CON group, which was an unexpected result. It is known that there are interaction effects between Se and Cu on absorption, retention, and distribution of these microminerals in the animal organism [68,69]. In addition, in our case, the form of presentation of selenium in both rations was different, which could affect the fractionation of these microminerals in the tissues and their excretion, and consequently on the milk and dairy products. Although, for the copper level, we only found significant differences in cheese. On the other hand, manufacturing process could affect Cu concentration [70], but in our experiment, this procedure was similar for both treatments. More studies must be done to clarify these possible effects.

In general, the texture profile of cheese did not show differences between treatments, except in terms of elasticity, which was slightly lower in the CON group. This could be due to the numerically greater amount of calcium found in this cheese, as Outinen and Rantamaki [71] found less elasticity in cheeses with higher calcium contents. Regarding the cheese sensory profile, despite finding a lower salty taste in the IOSe cheese, the overall acceptability was not affected. This is remarkable, as it indicates that inorganic plus organic Se supplementation did not influence the consumer’s acceptance of cheese.

## 5. Conclusions

We found that the effect of inorganic plus organic Se (as sodium selenite plus Se yeast) supplementation of feeding dairy cows at a moderate level (within the range of European Union legislation and with a wide safety margin) was more effective than sodium selenite, increasing milk Se concentration of dairy cows by 29.7% without altering its general metabolic status, and even decreasing the total oxidant status, and showing no deterioration in terms of overall milk quality and yield. However, this increase of Se was not observed in yogurt with inorganic plus organic Se supplementation. In fresh cheese obtained from sodium selenite plus Se yeast supplementation, there was 38.2% more Se compared to cows fed with an inorganic Se supplementation, achieving Se levels that are labelable as a “source of selenium”, without harming the quality of the product.

## Figures and Tables

**Table 1:** Ingredients and composition of the basal total mixed ration.

Item	Content
Ingredients, g/kg DM ^1^	
**Forage**	
Alfalfa hay	475.0
Barley straw	47.5
**Concentrate**	
Corn grain	177.0
Barley grain	99.5
Dried beet pulp	94.5
Corn gluten feed	36.7
Soybean meal (47% Crude Protein)	35.5
Palm oil	17.7
Sodium bicarbonate	7.8
Monocalcium phosphate	3.5
Sodium chloride	2.9
Vitamin-mineral premix ^2^	2.4
Calculated composition ^3^	
Crude Protein (g/kg DM)	156.0
Starch (g/kg DM)	232.7
Neutral Detergent Fiber (g/kg DM)	381.7
Net Energy for Lactation (MJ/kg DM)	6.52
Ca (g/kg DM)	11.8
P (g/kg DM)	3.9
Se (mg/kg DM)	0.240

^1^ DM = Dry Matter. ^2^ Provided (per kg of diet DM): vitamin A, 7992 IU; vitamin D_3_, 2088; vitamin E, 32 mg; vitamin B_12_, 0.0029 mg; niacin, 5.76 mg; biotin, 0.0019 mg; manganese, 24 mg; zinc, 57.6 mg; iron, 3.64 mg; copper, 13.2 mg; iodine, 0.96 mg; cobalt, 0.12 mg; and selenium, 0.240 mg, as sodium selenite in control diet (CON); and 0.144 mg of Se (as sodium selenite) plus 0.096 mg as organic selenium, in inorganic plus organic selenium diet (IOSe). ^3^ Estimated composition was calculated according to FEDNA [32].

**Table 2:** Analyzed composition of forages, concentrates, and total mixed rations of the experimental diets.

Item	Alfalfa Hay	Barley Straw	CON Concentrate ^1^	IOSe Concentrate ^2^	CON Ration ^3^	IOSe Ration ^4^
Analysed composition (g/kg DM ^5^)						
Ash	111.7	78.5	55.3	50.3	83.2	80.8
Ether Extract	22.1	17.4	22.8	23.0	22.2	22.3
Crude Protein	175	40.3	118	124	142	144
Neutral Detergent Fiber	546	785	196	199	389	390
Acid Detergent Fiber	394	505	49.8	50.2	235	235
Lignin Acid Detergent	91.2	91.6	40.5	41.7	67.0	67.6
P	2.3	0.2	5.3	5.5	3.6	3.7
Ca	14.5	3.2	7.4	5.7	10.5	9.7
Se (mg/kg)	0.066	0.042	0.382	0.423	0.205	0.225

^1^ CON Concentrate = control concentrate with inorganic Se. ^2^ IOSe concentrate = experimental concentrate with inorganic plus organic Se. ^3^ Total mixed ration with CON concentrate. ^4^ Total mixed ration with IOSe concentrate. ^5^ DM = Dry Matter.

**Table 3:** Effect of feeding inorganic plus organic selenium supplement on the mineral (Se, P, Ca, Cu, and Zn) and hemoglobin (Hb) composition, and the glutathione peroxidase (GSH-Px) activity, in whole blood of dairy cows at 0, 21, and 49 days of experiment.

Item		CON Ration ^1^		IOSe Ration ^2^			SEM ^3^	*p*-Value		
	T_0_^4^	T_21_	T_49_	T_0_	T_21_	T_49_		Ration (R)	Day (D)	R × D
Se (µg/L)	135.7	121.4	129.7	128.3	129.9	158.6	9.39	0.222	0.605	0.081
P (mg/100 g)	17.4	18.4	17.6	18.3	20.3	19.2	0.39	0.236	0.086	0.701
Ca (mg/100 g)	7.57	6.61	6.80	7.35	7.21	7.02	0.257	0.275	0.723	0.653
Cu (mg/100 g)	0.075	0.091	0.074	0.069	0.067	0.067	0.003	0.789	0.079	0.875
Zn (mg/100 g)	0.212	0.206	0.180	0.248	0.249	0.208	0.009	0.002	0.079	0.682
Hb (g/dL)	10.5	10.5	10.1	11.2	10.7	10.6	0.14	0.129	0.058	0.566
GSH-Px (U/L)	20,611	24,812	26,448	21,937	23,821	26,815	713.9	0.872	<0.001	0.377
GSH-Px (U/g Hb)	196.7	236.3	259.3	195.7	221.1	252.7	5.96	0.534	<0.001	0.547

^1^ Total mixed ration with inorganic Se. ^2^ Total mixed ration with inorganic plus organic Se. ^3^ SEM = standard error of the mean (*n* = 10 per treatment). ^4^ T_n_ = number (*n*) of days after starting the experiment.

**Table 4:** Effect of feeding inorganic plus organic selenium supplement on the biochemical profile and oxidative markers in the plasma of dairy cows at 0, 21, and 49 days of experiment.

Item	CON Ration ^1^			IOSe Ration ^2^			SEM ^3^	*p*-Value		
	T_0_^4^	T_21_	T_49_	T_0_	T_21_	T_49_		Ration (R)	Day (D)	R × D
Cholesterol (mg/dL)	223.6	264.5	265.4	230.2	259.7	282.8	12.71	0.805	<0.001	0.512
TG (mg/dL) ^5^	10.6	9.5	38.0	11.8	10.9	17.0	3.96	0.449	0.118	0.365
NEFA (mmol/L) ^6^	0.051	0.029	0.027	0.064	0.029	0.036	0.033	0.097	0.218	0.150
β-BHA (mmol/L) ^7^	0.400	0.331	0.261	0.384	0.448	0.375	0.015	0.036	0.004	0.008
Glucose (mg/dL)	64.6	66.0	66.2	64.6	66.4	63.6	0.77	0.650	0.829	0.386
Urea (mg/dL)	28.2	24.4	27.8	26.9	27.6	29.7	0.76	0.411	0.254	0.130
Total proteins (g/dL)	7.29	7.42	7.38	7.22	7.00	7.38	0.105	0.458	0.151	0.686
TOS (µmol/L) ^8^	24.6	17.8	25.3	15.2	18.6	15.1	1.187	0.018	0.932	0.921
TAC (mmol/L) ^9^	0.498	0.503	0.475	0.428	0.461	0.487	0.023	0.472	0.579	0.221

^1^ Total mixed ration with inorganic Se. ^2^ Total mixed ration with inorganic plus organic Se. ^3^ SEM = standard error of the mean (*n* = 10 per treatment). ^4^ T_n_ = number (*n*) of days after starting the experiment. ^5^ TG = Triglycerides. ^6^ NEFA = non-esterified fatty acid. ^7^ β-BHA = β-hydroxybutyrate. ^8^ TOS = Total oxidant status. ^9^ TAC = Total antioxidant capacity.

**Table 5:** Effect of feeding inorganic plus organic selenium supplement on milk yield, composition, and pH of milk of dairy cows at 0, 21, and 49 days of experiment.

Item	CON Ration ^1^			IOSe Ration ^2^			SEM ^3^	*p*-Value		
	T_0_^4^	T_21_	T_49_	T_0_	T_21_	T_49_		Ration (R)	Day (D)	R × D
Milk yield (L/d)	30.9	33.4	29.4	27.1	31.4	29.4	1.771	0.583	0.770	0.216
Milk composition										
Fat (g/100 g)	4.56	4.31	3.58	4.05	4.56	3.54	0.115	0.657	0.019	0.423
Protein (g/100 g)	3.25	3.02	3.08	3.41	3.18	3.05	0.035	0.199	0.027	0.395
Lactose (g/100 g)	4.77	4.72	4.80	4.95	4.68	4.81	0.019	0.244	0.026	0.130
Ca (mg/100 g)	118	113	119	122	113	119	3.27	0.838	0.809	0.460
P (mg/100 g)	85.4	81.3	91.1	86.3	83.6	87.7	1.69	0.981	0.132	0.360
Zn (mg/100 g)	0.447	0.449	0.418	0.456	0.442	0.412	0.013	0.958	0.047	0.655
Cu (mg/100 g)	0.007	0.003	0.003	0.006	0.004	0.004	0.000	0.675	<0.001	0.120
Se (µg/100 g)	1.54	1.33	1.26	1.64	1.94	1.80	0.047	0.001	0.789	0.167
Milk pH	6.61	6.79	6.65	6.64	6.67	6.62	0.023	0.418	0.611	0.177

^1^ Total mixed ration with inorganic Se. ^2^ Total mixed ration with inorganic plus organic Se. ^3^ SEM = standard error of the mean (*n* = 10 per treatment). ^4^ T_n_ = number (*n*) of days after starting the experiment.

**Table 6:** Effect of feeding inorganic plus organic selenium supplement on the physicochemical and mineral composition of yogurt.

Item	CON Ration ^1^	IOSe Ration ^2^	SEM ^3^	*p*-Value
Proximate composition and pH				
Protein (g/100 g)	5.71	7.91	0.737	0.148
Fat (g/100 g)	2.15	1.49	0.269	0.261
pH	4.53	4.71	0.070	0.226
Mineral composition				
Ca (mg/100 g)	214.8	201.4	12.4	0.045
P (mg/100 g)	170.7	164.1	8.97	0.184
Zn (mg/100 g)	0.874	0.848	0.038	0.650
Cu (mg/100 g)	0.422	0.410	0.116	0.877
Se (μg/100 g)	3.86	3.56	0.418	0.538
Physical characters				
Syneresis (g/100 g)	11.50	14.10	1.779	0.527
Colorimetric parameters ^4^				
L*	85.44	85.11	1.166	0.907
a*	−2.97	−3.23	0.104	0.252
b*	5.37	5.61	1.455	0.944

^1^ Total mixed ration with inorganic Se. ^2^ Total mixed ration with inorganic plus organic Se. ^3^ SEM = standard error of the mean (*n* = 3 yogurts × 3 batch for each treatment). ^4^ L* = lightness index, a* = red index, b* = yellow index.

**Table 7:** Effect of feeding inorganic plus organic selenium supplement on the physicochemical and mineral composition of cheese.

Item	CON Ration ^1^	IOSe Ration ^2^	SEM ^3^	*p*-Value
Proximate composition				
Dry extract (g/100 g)	34.65	30.68	0.869	0.013
Protein (g/100 g)	15.36	14.74	0.539	0.588
Fat (g/100 g)	12.71	9.07	2.786	0.101
Mineral composition				
Ca (mg/100 g)	559.4	505.0	21.1	0.062
P (mg/100 g)	351.0	323.1	13.44	0.110
Zn (mg/100 g)	2.81	2.83	0.25	0.923
Cu (mg/100 g)	1.84	0.70	0.03	<0.001
Se (μg/100 g)	6.00	8.29	0.83	0.001
Physical characters				
Cheese yield	17.1	20.2	1.15	0.209
Colorimetric parameters ^4^				
L*	89.08	89.67	0.326	0.427
a*	−2.62	−2.60	0.046	0.857
b*	7.27	8.06	0.338	0.287
Texture profile				
Hardness (N)	10.01	8.48	0.638	0.273
Cohesiveness (dimensionless)	1.20	1.21	0.031	0.974
Adhesiveness (N s)	−0.70	−0.23	0.177	0.192
Elasticity (mm)	1.21	1.23	0.004	0.009
Chewiness (N mm)	12.28	10.25	0.851	0.241

^1^ Total mixed ration with inorganic Se in the vitamin-mineral premix of concentrate. ^2^ Total mixed ration with inorganic plus organic Se in the vitamin-mineral premix of concentrate. ^3^ SEM = standard error of the mean (*n* = 3 cheeses x 3 batch for each treatment). ^4^ L* = lightness index, a* = red index, b* = yellow index.
